# How Can Agricultural Corporate Build Sustainable Competitive Advantage through Green Intellectual Capital? A New Environmental Management Approach to Green Agriculture

**DOI:** 10.3390/ijerph18157900

**Published:** 2021-07-26

**Authors:** Chulin Pan, Yufeng Jiang, Mingliang Wang, Shuang Xu, Ming Xu, Yixin Dong

**Affiliations:** 1 College of Biological and Agricultural Engineering, Jilin University, 5988 Renmin Street, Changchun 130022, China; pancl@jlu.edu.cn (C.P.); jlu_wml@163.com (M.W.); xushuang_jlu@163.com (S.X.); 2 School of Public Administration, Changchun University of Technology, 2055 Yan’an Street, Changchun 130012, China; 3 Yatai School of Business Administration, Jilin University of Finance and Economics, 3699 Jingyue Street, Changchun 130117, China; xuming@jlufe.edu.cn (M.X.); iamdongyixin@sina.com (Y.D.)

**Keywords:** green intellectual capital, green innovation, agricultural corporate sustainable competitive advantage, green agriculture

## Abstract

Based on natural resource-based theory, this study constructed a relational model between green intellectual capital, green innovation, and an agricultural corporate sustainable competitive advantage. The samples included a total of 341 agricultural companies in China, and multiple regression methods are used for the analysis. The results showed that green product innovation and green process innovation had a mediation effect between green human capital, green structural capital, green relational capital, and the sustainable competitive advantage of agricultural corporate. Beyond the simple moderation effect, a new integrated moderated-mediation effect model was established. It was shown that environmental leadership, green organizational identification, and green dynamic capability had different moderated-mediation effects under different conditions. The study is expected to close the previous research gaps and insufficiency in agricultural corporate environmental management and green agricultural. The empirical results and conclusions bring enlightenment and meaningful theoretical guidance to managers, researchers, practitioners, and policy makers in the green and sustainable development of agricultural corporates. The new environmental management path can help agricultural corporates conduct green innovation effectively, adapt to the green agricultural products market, and achieve sustainable competitive advantage. Ultimately, this will help to accelerate the development of green agriculture.

## 1. Introduction

Global warming, desertification, haze, and other environmental problems have become the focus of all industries in the process of global economic development [1]. Against the background of excessive resource consumption, serious environmental pollution, and increasing demand for green agricultural products, it is inevitable and necessary for agricultural development to take a green and sustainable development path. It is urgent to ease the tension between agricultural economic development and environmental bearing capacity. “Our Common Future” published by the World Council for Environment and Development (WCED) in 1987 puts forward the concept of “sustainable development”, pointing out that the earth’s existing resources and energy are far from meeting the needs of human development, and environmental protection has a positive and far-reaching impact on sustainable development [2].

As the most important microeconomic subject for the green development of agriculture, agricultural enterprises have certain particularities compared with enterprises in other industries. The people regard food as heaven, and food safety is very important. Agricultural production is greatly affected by the natural environment. Agricultural products are closely related to the natural environment and human life. However, pollution is common in the process of agricultural development, and the agricultural product-processing industry especially has a negative impact on the environment [3]. With the improvement of consumers’ awareness of environmental protection and the increasing demand for green food, organic food, and other green agricultural products, the demand for ecological and environmental protection and high-quality and safe green agricultural products in the production process of agricultural products enterprises is also increasing. The “green” and “sustainability” of agricultural enterprises have become national and even global expectations [4].

Existing scholars have studied the relationship between environmental behavior and competitive advantage from the perspective of sustainable development, believing that environmental management and green innovation are no longer the “protection umbrella” for enterprises to avoid violations, but the effective way for enterprises to achieve sustainable development [5,6]. More and more entrepreneurs’ cognition of environmental management has changed from the perspective of obedience and commitment to the strategic perspective of long-term sustainable development of enterprises [7]. Natural resource-based view (NRBV) theory constructs a model of economic and environmental and sustainable development of enterprises [8]. Most scholars believe that environmental protection can bring competitive advantages to enterprises, and enterprise environmental management is no longer the “umbrella” for enterprises to avoid violating environmental regulations, but the key for enterprises to obtain sustainable competitive advantages [1,9,10].

However, the existing literature lacks research on the environmental management behavior of agricultural enterprises, although this research is very important. Although the existing studies have explored the positive influence of enterprise environmental management on enterprise competitive advantage [6,11,12,13], they have not conducted an in-depth discussion from the perspective of internal and external resources and capabilities of enterprises based on the theory of natural resource foundation. More importantly, the existing studies have proved the issue of “whether or not” but have not explored the issue of “how” thoroughly.

The resource-based theory holds that the sustainable development of enterprises cannot be separated from strategic resources [14]. From the perspective of resources and ability, NRBV focuses on the relationship between enterprise behavior and the natural environment and constructs an economic model of sustainable development between enterprises and the environment from the perspective of resources and capabilities [8]. In the era of the knowledge economy, intellectual capital has become the most important intangible asset of modern enterprises and an important strategic resource for the sustainable development of enterprises [15,16]. According to the view of intellectual capital theory, the accumulation and management of green intellectual capital can bring a competitive advantage to enterprises through establishing a good relationship with nature [9]. Green human capital, green structural capital, and green relational capital are closely related to the sustainable development of enterprises [17].

Green agriculture is inseparable from innovation [4]. Green innovation aims to reduce the negative impact of products or production processes on the environment and coordinate the economic effect and ecological effect of enterprises [18,19]. Compared with general innovation, green innovation has “double externalities”, that is, in addition to having the same positive spillover effects as other general innovations, green innovation will generate positive externalities by reducing the cost of the external environment and reduce the negative externalities [20]. Green innovation can compensate for the costs caused by environmental regulation, enabling enterprises to obtain “Innovation offsets” and “First Mover Advantage”. This behavior cannot be separated from the support of enterprise resources, and intellectual capital is one of the most important resources for agricultural enterprise innovation [21].

However, there are few scholars who have explored the issue of green intellectual capital and green innovation and sustainable development of agricultural enterprises from the perspective of environmental management, which await further scholarly investigation [9,17]. In addition, the existing studies still do not solve the problem of how agricultural enterprises can transform “green” capital into the source of sustainable competitive advantages, nor do they explain in detail how various types of green intellectual capital influence green product innovation and green process innovation to give agricultural enterprises a sustainable competitive advantage. Previous studies did not study green intellectual capital in different dimensions, nor did they explore their different impact effects on green innovation.

Although “leadership”, “organizational identification”, and “dynamic ability” have been well studied in the academic circle and widely applied in the management circle, few studies focus on their combination with natural environmental factors and ignore their application in the field of enterprise environmental management. Agricultural enterprises’ environmental behaviors require the guidance and encouragement of leaders, the recognition and support of organization members, as well as strong adaptability and a quick response to the green environment [22,23,24]. Existing studies lack the comprehensive consideration of static resources and dynamic capabilities and ignore the important role of green dynamic capabilities in environmental management and green innovation. Moreover, they have not deeply explored the boundary conditions of sustainable competitive advantage formation from the perspective of the natural resource-based view and have not formed a relatively complete enterprise sustainable development model in the field of environmental management.

In the context of resource scarcity, ecological damage, and under the strict environmental laws and increasing environmental pressure of stakeholders, it is particularly necessary for agricultural enterprises to combine sustainable development with the problems of natural resources and the environment [25]. Can agricultural enterprises establish sustainable competitive advantages through the accumulation and application of green intellectual capital and green innovation? How can different kinds of green intellectual capital and green innovation be applied to make different environmental management strategy choices? What contingency factors affect green innovation and the competitive advantage of agricultural corporate? This study will try to answer these questions by exploring the influence mechanism of green human capital, green structural capital, and green relational capital on green product innovation, green process innovation, and sustainable competitive advantage. The research will integrate environmental leadership, green organizational identification, and green dynamic capability into the overall framework and propose an integrated moderated-mediation effect model that three moderating variables changed simultaneously. In addition, the different influence paths will be compared and analyzed.

The study found that green product innovation and green process innovation had a mediation effect between green human capital, green structural capital, green relational capital, and the sustainable competitive advantage of agricultural corporate. Environmental leadership, green organizational identification, and green dynamic capability had positive moderation effects, and they had different moderated-mediation effects under different conditions. The study is expected to close the previous gaps and insufficiency and draw a meaningful research conclusion and the management enlightenment and bring theoretical guidance to agricultural enterprises in the process of green intellectual management, green innovation, and agriculture green sustainable development.

The remainder of the paper is structured as follows: The literature review is conducted and a theoretical framework is presented in Section 2. Section 3 presents materials and methods. Empirical results are presented in Section 4. Section 5 and Section 6 provide discussions, some key conclusions, limitations, and further research.

## 2. Literature Review and Theoretical Framework

### 2.1. Green Intellectual Capital and Agricultural Corporate Sustainable Competitive Advantage

Porter (1995) put forward the importance of sustainability of competitive advantage [1]. Sustainable competitive advantage ensures the sustainable and long-term dominant position of the enterprise [26]. Jones et al. (2018) established a sustainable competitive advantage model from the perspective of stakeholder management based on the resource-based view theory [27]. Sustainable competitive advantage is not limited to a certain calendar time but transcends the concept of a certain point in time [28]. It means that an enterprise has certain special resources or capabilities that are not quickly imitated and replaced by competitors. Most scholars who support the theory of endogenous competitive advantage believe that the sustainable competitive advantage of enterprises comes from the heterogeneous resources within enterprises [29].

The natural resource-based view (NRBV) focuses on the relationship between enterprises and the natural environment and constructs a sustainable development economic model between enterprises and the environment from the perspective of resources and capabilities [8]. Ahmad (2015) proposed that the sustainable competitive advantage of enterprises has three attributes: Economic sustainability, environmental sustainability, and social sustainability, which comes from the intellectual capital accumulated by enterprises through knowledge management [26]. Green intellectual capital is put forward under the serious environment pollution background and is a kind of intellectual capital that is related to enterprise environmental management [9]. Chang and Chen (2012) divided green intellectual capital into green human capital, green structural capital, and green relational capital [17]. According to this classification, the following analysis has been conducted.

First, in terms of human capital, Neves and Borges (2018) established an environmental management model of green human resource management based on NRBV theory [30]. The sustainable competitive advantage of agricultural enterprises requires green human resources and efficient green human resource management [31]. Employees’ knowledge about environmental protection and green technology, as well as skills and commitment to environmental protection and green innovation, are the basic guarantee for agricultural enterprises to obtain sustainable competitive advantages [32].

Second, in terms of green structural capital, there are examples such as an environmental management system, environmental protection enterprise culture, environmental protection commitment, a knowledge management system, a green information technology system, a green logo, a green brand, and a green corporate image. For example, environment-oriented agricultural enterprises integrate their environmental issues into corporate culture, decision-making, and the operation system are all important resources of sustainable competitive advantages for agricultural enterprises [9,33].

Third, in terms of green relationship capital, the enterprise establishes long-term relationships with suppliers, customers, partners, investors, offering green products and services such as trust, commitment, and cooperation, making upstream and downstream products’ environmental standards, sharing environmental knowledge, which can not only promote the enterprise image, increase customer satisfaction and loyalty, but still increase the trust of the stakeholders and promote the competitive advantage [34].

In the process of establishing a sustainable competitive advantage, agricultural enterprises cannot do without green intellectual capital that is valuable, scarce, unique, difficult to imitate, and difficult to replace. Green intellectual capital is the key strategic resource for agricultural enterprises to obtain sustainable competitive advantage and sustainable development in the process of environmental management. Thus, based on NRBV theory, we proposed the following hypothesis.

**Hypothesis** **1.** *Green human capital (H1a), green structural capital (H1b), and green relational capital (H1c) have a positive influence on the sustainable competitive advantage.*

### 2.2. The Mediation Effect of Green Innovation

Green innovation is the sum of new ideas and behaviors applied by agricultural enterprises in the process of environmental management for production or a series of innovative behaviors in the production process [35]. Some scholars define green innovation in a broader sense, that is, all innovation behaviors that can reduce the negative impact on the environment belong to green innovation [36]. De Marchi (2012), Guoyou et al. (2013), and other scholars, based on the innovation objects, hold that green innovation includes the innovation of the green concept and design of the product itself, as well as the innovation of resource conservation, pollution prevention and control, waste recycling, and other behaviors in the production process [19,37,38,39]. Most scholars divide green innovation into green product innovation and green process innovation [9,23].

Papagiannakis et al. (2014) argued that the accumulated resources and capabilities of agricultural enterprises in the past could stimulate more environmental behaviors and enable agricultural enterprises to choose higher-level environmental behaviors, such as green innovation [40]. Lin and Ho (2008) found that the quality of human resources and organizational incentives had a significant positive impact on agricultural enterprises’ intentions to green innovation [41]. Talke et al. (2006) believe that the development of knowledge and ability plays an important role in enterprise innovation [42]. Innovation behavior cannot be separated from the support of enterprise resources. Intellectual capital is one of the most important resources for enterprise innovation to enhance its innovation ability [21].

In the process of green innovation, agricultural enterprises need employees to provide knowledge, experience, and skills of environmental management. Green human capital is the basic element of green innovation in agricultural enterprises [30]. The existing environmental management system of agricultural enterprises can help them break through the original environmental standards and take the initiative to innovate. Corporate green culture creates a good atmosphere for corporate green innovation. Shu et al. (2020) believed that green management has a stronger positive effect on innovation than on financial performance [43]. Agricultural enterprises can promote green product innovation and process innovation with green structure capital. The establishment of green cooperative relations between agricultural enterprises and suppliers or strategic partners will facilitate the sharing of green knowledge, accelerate the process of green innovation, and promote collaborative innovation [9]. In particular, partnerships with universities and research institutions will promote the development of green products and green technologies.

According to innovation compensation theory, green innovation can promote agricultural enterprises to improve product quality and the production process, as well as boost productivity, increase the resources utilization ratio, and save energy. Green innovations also have a positive effect on a firm’s environmental performance [18,44]. Therefore, green innovation has “double externalities”, which can not only reduce negative external effects, but also bring positive spillover effects of “innovation compensation” and the “first-mover advantage”. Yao et al. (2021) confirmed the positive impact of product innovation and green process innovation on brand equity [45]. Porter and Van der Linde (1995) pointed out that there is an “environmental premium” in green market transactions [1], that is, consumers tend to pay more expensive fees for environment-friendly products [46]. Agricultural enterprises can not only demand higher product premiums from consumers through green innovation to make up for environmental management costs, but also establish barriers to entry for industry competitors [47]. Therefore, the sustainable competitive advantage of agricultural enterprises comes not only from the green intellectual capital, but also from the green product innovation and process innovation through the accumulation and application of green intellectual capital. Thus, we proposed the following hypothesis.

**Hypothesis** **2.** *Green product innovation (H2a) and green process innovation (H2b) have a mediation effect between green intellectual capital and sustainable competitive advantage*.

### 2.3. The Moderation Effect of Environmental Leadership

Leaders’ environmental protection values and attitudes towards environmental issues affect enterprises’ enthusiasm in implementing environmental strategies [48,49]. The vision and policy of environmental protection established by the leader in the enterprise determines the level of environmental issues in the overall strategy of the enterprise, thus affecting the enterprise’s environmental behavior [50].

Environmental leadership involves implementing factors concerning environmental protection and sustainable development, which will influence employees to carry out work tasks without threatening the natural environment, reduce the negative impact on the environment in production operations, and make changes that benefit the environment at work [22]. This type of leadership reflects the corporate leader’s concern for environmental protection and sustainable development [51]. The stronger the environmental leadership is, the more the organization can be motivated to realize the vision of green and sustainable development [52]. Environmental leadership embodies the characteristics of transformational leadership and guides agricultural enterprises to actively carry out green innovation [53,54].

The process of agricultural enterprises applying green intellectual capital for innovation is influenced by environmental leadership. First of all, environmental leadership runs through the whole dynamic process of individuals influencing others to implement environmental management and environmental protection [55]. Environmental leadership influences individual consciousness and behavior and mobilizes organization members to identify and strive to realize the enterprise’s long-term vision of ecological and sustainable development [52]. The level of environmental leadership affects the enthusiasm of agricultural enterprises to adopt green innovation, and managers can motivate employees with environmental technologies to participate in enterprise environmental behavior and green innovation [9].

Secondly, effective environmental leaders pay more attention to ecologically centered values under environmental commitment and ethical considerations, as well as paying more attention to the application of environmental resources and incentives for green innovation. Organizational leaders not only affect employees’ attitude and commitment, but also affect organizational performance and other organizational outputs, including environmental performance, degree of greening, and efficiency effect of green change [56]. Agricultural enterprises with strong environmental leadership are more likely to carry out green innovation through the application of green structural capital.

Finally, environmental leadership can enhance strategic communication, knowledge sharing, and cooperation between agricultural enterprises and customers, suppliers, or other partners [57]. When environmental leadership is strong, agricultural enterprises are more likely to make use of the green relationship capital established with stakeholders for enterprise innovation [58]. Environmental leadership enables agricultural enterprises to build close relationships with suppliers or partners, learn from each other, and apply green technologies and capabilities, share environmental information and resources, and apply them to green innovation to improve innovation performance. Thus, we proposed the following hypothesis.

**Hypothesis** **3.** *Environmental leadership has a positive moderation effect between green intellectual capital and green product innovation (H3a) or green process innovation (H3b).*

### 2.4. The Moderation Effect of Green Organizational Identification

From the psychological point of view, identification refers to a specific emotional connection, and it is an explanatory plan jointly made by the members of the organization, giving specific meaning to their behaviors and choices [59]. Organizational identification is a set of beliefs about what is core, enduring, and different [60]. Although organizational identification has been widely discussed in previous studies, few studies have focused on the natural environmental factors in organizational identification and applied organizational identification to the field of corporate green innovation.

Based on the dual demands of corporate economic development and corporate social responsibility fulfillment, the framework of organizational identification is inseparable from the consideration of environmental protection. According to the theory of organizational identification, organizational green behavior is embedded in the cognitive and emotional foundation of organizational members, which makes green organizational identification closely related to organizational environmental strategic behavior. Fernández et al. (2003) proposed that when an enterprise identifies with its own environmental behavior, the enterprise will integrate this emotional connection into its management behavior and motivate the enterprise to carry out environmentally friendly corporate strategic behavior [61].

Green organizational identification refers to the common beliefs about environmental management and green innovation that bind individuals and organizations together. Chen (2011) believes that green organizational identification is an organizational identification mode about environmental management and green innovation jointly established by organization members that give significance to environmental protection behaviors [55]. Green organizational identification helps members clearly understand the relationship between the organization’s environmental protection objectives and actions and build a shared interpretation model based on understanding and mining the profound meaning of surface behaviors. Through the structural equation model, Chen and Chang (2013) verified that green organizational identification has a positive effect on green intangible assets and green competitive advantages [23]. According to organization identity theory, green organizational identification is the key factor of environmental management [62]. Green organizational identification has a positive effect on enterprise environmental behavior such as green innovation [63,64].

Sharma (2000) found that the integration of organizational identification can integrate and summarize different knowledge structures and promote the generation of organizational innovation behaviors [65]. When the enterprise has a sense of identity towards environmental problems, the enterprise will actively develop clean energy and adopt clean technology in strategic practice to protect the natural environment. The green organizational identification of agricultural enterprises can enhance the corporate social responsibility and influence the innovation behavior of agricultural enterprises by integrating knowledge and behavior selection. The stronger this sense of identity is, the more the agricultural enterprises can apply green human capital, green structural capital, and green relational capital to innovatively integrate green elements in product design, packaging, production, and other processes to carry out green product innovation and green process innovation. Thus, we proposed the following hypothesis.

**Hypothesis** **4.** *Green organizational identity has a positive moderation effect on green intellectual capital and green product innovation (H4a) or green process innovation (H4b).*

### 2.5. The Moderation Effect of Green Dynamic Ability

With the acceleration of knowledge spillover and technological progress, as well as the rapidly changing green consumer market, the comparative advantage of green resources may dissipate, and the static analytical competitive advantage is challenged [66]. Traditional resource capability theory is limited to static analysis from the inside out, which cannot give agricultural enterprises an answer to how to obtain a sustainable competitive advantage in the rapidly changing and unpredictable dynamic market. In the fast and changeable global market competition environment, those agricultural enterprises with keen insight and a quick reaction ability can effectively coordinate and allocate internal and external resources and obtain a sustainable competitive advantage.

Scholars regard dynamic capability as the key factor that affects the competitive advantage of enterprises [67,68]. The “dynamic” of dynamic capability originates from the uncertainty of the external environment, which brings both opportunities and threats to agricultural enterprises [69]. Agricultural enterprises need to identify and grasp opportunities for resource reorganization [70]. Green dynamic ability refers to the ability of agricultural enterprises to make internal and external adjustment related to environmental management in time according to the dynamic development and change of the environment to adapt to the environmental protection policy orientation and the rapidly changing green market demand [23]. This ability enables agricultural enterprises to recombine internal and external green resources through organizational learning, and to establish a new enterprise environmental strategy convention that breaks through the dependence of the original environmental strategy path [71]. Green dynamic capabilities include the ability to identify opportunities for new green quickly, the ability of identifying and developing new green knowledge or green technology, and green innovation ability [72].

Chen and Chang (2013) divided green dynamic ability into green environment adaptation ability, green resource integration ability, organization learning and absorption ability, and green change ability [23]. First, only by quickly identifying stakeholders’ requirements for cleaner production and consumers’ demand for green products, and making strategic, operational, or organizational adjustments to the environment in a timely manner, can agricultural enterprises be promoted to bring sustainable competitive advantages through green product innovation and process innovation.

Second, in the process of green innovation, enterprises need to identify, dig, acquire, and apply green resources from different levels [73,74]. Both environmental protection knowledge and green information technology are important green resources for agricultural enterprises, which need to be updated and reconfigured constantly to respond to the changes of the external environment. As knowledge spirals within the enterprise, green innovation can be generated and bring more benefits. The stronger the dynamic ability, the more efficient the knowledge use and integration, the higher the probability of innovation success, and the more lasting the competitive advantage of agricultural enterprises.

Third, in addition to making effective use of existing environmental knowledge, enterprises’ green innovation also needs to identify, acquire, analyze, and understand new environmental knowledge, process, digest, and apply new environmental knowledge and technologies [75,76]. Innovation by reference to knowledge is also included [77]. The establishment of green knowledge sharing and transfer mechanism, the effective dissemination of green knowledge and information, and the learning and training of environmental knowledge have a positive impact on the transformation of green innovation into a sustainable competitive advantage of agricultural enterprises [78].

Last, according to the dynamic ability theory and Schumpeter’s innovation-based competition theory, agricultural enterprises should carry out a green revolution according to the market demand for green products and the competitive situation of the green market to obtain sustainable competitive advantage [79]. Such green “creative destruction” can enable agricultural enterprises to make more rapid responses and decisions in the face of environmental pressure from stakeholders and changing green market demands, improving the success rate of enterprise product and process innovation. Therefore, green dynamic capability can make the competitive advantage brought by green product innovation and green process innovation more sustainable. Thus, we proposed the following hypothesis.

**Hypothesis** **5.** *Green dynamic capability has a positive moderation effect between green product innovation and sustainable competitive advantage (5a), green process innovation, and sustainable competitive advantage (5b*).

### 2.6. An Integrated Moderated-Mediation Effect Model

**Figure 1:**
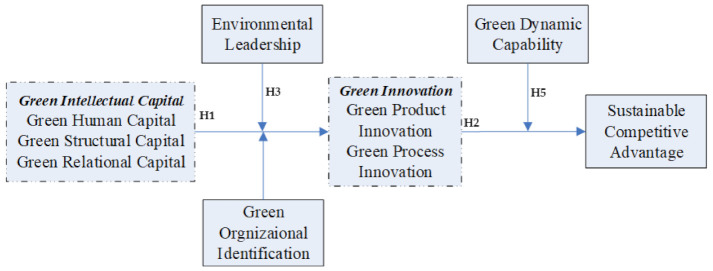
Conceptual model.

The study has proposed that green innovation has a mediation effect between green intellectual capital and agricultural corporate sustainable competitive advantage and environmental leadership, green organizational identity has a positive moderation effect between green intellectual capital and green innovation, and green dynamic capacity has a positive moderation effect between green innovation and agricultural corporate sustainable competitive advantage. Therefore, according to the mediating and moderation effect proposed by Edwards and Lambert (2007) [80], we believe that environmental leadership, green organizational identity, and green dynamic capacity also moderate the mediation effect [81]. The higher the environmental leadership, green organizational identity, and green dynamic capacity are, the higher the mediation effect of green innovation between green intellectual capital and agricultural corporate sustainable competitive advantage is. In this view, this study proposes an integrated mediation and moderation effect model (Figure 1). Hence, it is proposed that:

**Hypothesis** **6.** *Under higher environmental leadership, green organizational identity, and green dynamic capacity, the mediating effect of green innovation between green intelligence capital and agricultural corporate sustainable competitive advantage is higher*.

## 3. Materials and Methods

### 3.1. Data Collection and Sample

“Food and safety come as the first”. Agricultural products are related to the natural environment and human life. China is a big agricultural country. The environmental behavior of agricultural enterprises directly affects the environmental protection and the safety of agricultural products. However, in recent years, the pollution phenomenon is serious, especially as the agricultural product processing industry brings a negative impact on the environment. With the improvement of consumers’ awareness of environmental protection and the increasing demand for green food, pollution-free agricultural products marked with green environmental protection, organic food, and other green agricultural products, the requirements for agricultural enterprises to produce ecological environmental protection and high-quality and safe green agricultural products are also increasing day by day.

We conduct empirical research by means of questionnaire surveys. We selected several agricultural universities that have cooperative relationships with the department of agricultural economic management of the authors’ university in the teaching and research area. Our team had been visiting Jilin Agricultural University, Shenyang Agricultural University, Northeast Agricultural University, Nanjing Agricultural University, Zhejiang A&F University, and Fujian A&F University since September 2019, a total of six universities. We interviewed teachers majoring in economic management from the above universities, contacted MBA students working in agricultural enterprises or members of training courses for corporate executives, and obtained a list of local agricultural enterprises. Based on the report of the “Regional distribution of China’s top 500 agricultural enterprises by 2020”, northeast China, Yangtze River Delta, and Pearl River Delta, where agricultural production enterprises are concentrated, are selected as the main investigation areas. The three northeastern provinces have important agricultural production bases in China, the Yangtze River Delta and the Pearl River Delta are relatively concentrated centers of innovation and application, and there are also many agricultural production and processing enterprises.

The surveyed agricultural enterprises come from Changchun, Harbin, Shenyang, and other cities in Northeast China; Suzhou, Nanjing, Nantong, Hangzhou, Ningbo, and Wenzhou in the Yangtze River Delta region; and Guangzhou, Shenzhen, Zhuhai, and Huizhou in the Pearl River Delta region. It includes 9 industries: The food processing industry, the food manufacturing industry, the beverage manufacturing industry, the tobacco processing industry, the textile industry, the wood processing industry, the furniture manufacturing industry, the paper and paper products industry, and the rubber products industry. Due to the global COVID-19 pandemic encountered in this survey, face-to-face interviews have been cancelled since the middle of January 2020, and all interviews were replaced by telephone interviews and online questionnaires.

Based on the previous studies, this study designed the questionnaire and adjusted and modified the measurement items appropriately according to the Chinese context. In this study, data were collected by means of questionnaires, and the research objects were department managers or general managers of agricultural enterprises in the above regions. In order to improve the rate of questionnaire recovery and prevent emails from being automatically blocked, members of the research group called each agricultural product enterprise to explain the purpose of the study and the contents of the questionnaire and explained that the questionnaire was filled in anonymously to guarantee a degree of confidentiality of the questionnaire.

The author conducted a preliminary survey in three provinces in northeast China, and according to the results of the preliminary survey, repeatedly improved the setting of questions and the expression of questions in the questionnaire. Finally, 600 formal questionnaires were formed from 370 questionnaires that were recovered and the recovery rate was 61.67%. Twenty-nine incomplete and invalid questionnaires were excluded, and the valid questionnaires totaled 341. Table 1 summarizes the characteristics of the enterprises.

### 3.2. Variables and Measure

Our dependent variable is the agricultural corporate sustainable competitive advantage. Its measure was adopted from Ahmad (2015) [26], which contained 12 items from both financial and nonfinancial perspectives. Example items are “the customer loyalty is higher for green products or services, and many are regular and introduced customers,” “the green products and services make enterprises keep a high growth rate of sales revenue during a period of time,” “investors have a better evaluation of enterprise environmental protection behavior, and are willing to continue the investment,” and “The outstanding performance of enterprises in environmental protection attracts and retains talents.”

Our independent variables are green human capital, green structural capital, and green relational capital. There measures were adopted from Chen and Chang (2013) [23]. Green human capital contained 5 items, and example items are “the productivity and contribution of environmental protection of the employees in the firm is better than those of its major competitors,” and “the cooperative degree of team work about environmental protection in the firm is more than that of its major competitors”. Green structural capital contained 9 items, and example items are “the investments in environmental protection facilities in the firm are more than those of its major competitors,” and “the management system of environmental protection in the firm is superior to that of its major competitors”. Green relational capital contained 5 items, and example items are “the firm designs its products or services in compliance with the environmentalism desires of its customers,” and “the cooperation relationships about environmental protection of the firm with its upstream suppliers are stable”.

The mediator variables are green product innovation and green process innovation. These measures were adopted from Kam-Sing (2012) and Song and Yu (2018) [62,82]. Green product innovation contained 4 items, example items are “Enterprise chooses materials that consume the least energy and resources during product development and design,” and “Enterprise chooses materials with the least environmental pollution during product development and design”. Green process innovation contained 4 items, and example items are “Enterprise reduces the discharge of solids, water and other pollution in the production process,” and “Enterprise reduces the use of raw materials in the production process.”

The one moderator variable is environmental leadership. Its measure was adopted from Chen (2011) [55], which contained four items, and example items are “Enterprise’s leaders encourage organizations to establish a common vision of environmental values,” and “Enterprise’s leaders educate your employees about environmental protection regularly.” The one moderator variable is green organizational identity. Its measure was adopted from Chen, which contained 6 items, and example items are “the enterprise’s top managers, middle managers, and employees feel that the enterprise have formulated a well-defined set of environmental goals and missions,” and “the enterprise’s top managers, middle managers, and employees have a strong sense of the enterprise’s history about environmental management and protection”. The other moderator variable is green dynamic capacity. Its measure was adopted from Makkonen et al. (2014) and Chen and Chang (2013) [23,69], which contained 8 items, and example items are “Enterprise keeps abreast of consumer green demand and industry green technology changes, and take appropriate measures,” and “Enterprises continue to learn and absorb knowledge about environmental protection and green innovation.”

The control variables are enterprise scale and enterprise type. Delgado-Ceballos et al. (2012) found that enterprise size is also one of the factors affecting enterprise environmental behavior [83], while Huang et al. (2014) found that different types of enterprises have different environmental behaviors. The enterprise scale is represented by the number of employees [49]. Enterprise types are divided into state-owned agricultural enterprises and nonstate-owned agricultural enterprises measured by dummy variables.

## 4. Empirical Results

### 4.1. Measurement Validation

All variables were measured by the instruments previously developed and used worldwide. Before the questionnaires were distributed, the instruments were translated into Chinese and then into English to ensure consistency. According to the Chinese context and the purpose of this study, the questionnaire was modified appropriately. The instruments had also been pilot-tested on MBA students from Jilin university, Northeast normal university, and Jilin university of finance and economics to ensure that translations do not affect the validity and reliability of these measures.

We analyzed the reliability first, and Cronbach’s alpha coefficients were regarded as the judgment standard. The results are presented in Table 2. One item of green structural capital GIC11 did not meet the threshold. When we deleted it, the Cronbach’s alpha coefficient of the green structural capital increased, so we deleted it. All other constructs’ Cronbach ‘s alpha values were greater than 0.8. Deleting any item after parameter values did not increase this value. From Table 2, we know that the Cronbach’s alpha coefficients of the structures ranged from 0.818 (green relational capital) to 0.907 (green dynamic capability), which can be regarded as reliable because the three constructs were all above the acceptable threshold of 0.50 [84]. The reliability of the scale was high.

Since all the measuring instruments were based on western research, it was necessary to evaluate their validity in the Chinese context. We used confirmatory factor analysis (CFA) to assess the validity of all the instruments.

We developed a measurement structure of all the variables and covariated them in a single model. The nine-factor model produced the best fit with data (χ^2^/df = 2.947; comparative fit index (CFI) = 0.957; Tucker Lewis index (TL) = 0.943; root mean square error of approximation (RMSEA) = 0.073; standardized root mean square residual (SRMR) = 0.049). The eight-factor to one-factor models produced a poor fit with data. In the nine-factor model, the standardized factor loading was higher than 0.50 [85]. We also used the average variance extracted (AVE) method to analyze the convergent and discriminate validity of all nine latent variables [86]. The AVE values exceeded the recommended threshold of 0.50. In addition, the lowest composite reliability (CR) value was 0.874, which was higher than the suggested threshold of 0.70. Thus, all constructs have high convergent validity. For assessing discriminant validity, we used the most rigorous and powerful method, and the AVE square root was more than its correlation with any other latent variable. From Table 3 we could see that the square root of AVE by each construct was greater than their following correlations, and we concluded that these constructs were different from each other (see Table 3). Therefore, we could say the convergent and discriminate validity of the scale was high.

Second, we used a multivariate *t*-test to assess nonresponse bias by comparing early and late responses for all variables [87]. The nonsignificant results showed that nonresponse bias was not present. Moreover, we have taken some measures to make sure common method bias (CMB) is minimized. In order to reduce common method variance, all items in each of the constructs are randomized [88]. We performed Harman’s single factor test to assess if the CMB could be an issue. The 60 items in the questionnaire were loaded, and exploratory factor analysis was performed using non-rotating principal component analysis (NPCA). The KMO was 0.858. The result revealed the presence of all distinct factors with eigenvalues greater than 1 which account for 68.036% of the variance, and the first factor accounts for only 21.880% of the variance. Third, we checked all the correlations between items; there were no extremely high correlations between items, so common method variance was not problematic. Therefore, we concluded that the CMB was not an issue in this study.

### 4.2. Hypothesis Testing

This study used hierarchical multiple regression analysis to examine the effect of green intellectual capital on sustainable competitive advantage, considering the mediating role of green innovation and the moderating role of environmental leadership, green organizational identification, and green dynamic capability. We tested for collinearity by calculating the variance inflation factor (VIF) for each of the regression coefficients in the model. Values were all below the suggested cut-off threshold of 10 (ranged from 1.067 to 1.965), suggesting a limited threat of multicollinearity [89].

From Model 1 to Model 3 in Table 4, it can be seen that green human capital (standard β = 0.300, *p* < 0.001), green structure capital (standard β = 0.218, *p* < 0.001), and green relational capital (standard β = 0.290, *p* < 0.001) had a significant negative effect on sustainable competitive advantage. H1 was supported. From Model 4 it can be seen that green product innovation had a significant positive effect on sustainable competitive advantage (standard β = 0.195, *p* < 0.001), and the effect of green human capital on sustainable competitive advantage changed from 0.300 to 0.249 (*p* < 0.001). From Model 6 it can be seen that green product innovation had a significant positive effect on sustainable competitive advantage (standard β = 0.223, *p* < 0.001), and the effect of green structure capital on sustainable competitive advantage changed from 0.218 to 0.174 (*p* < 0.001). From Model 8 it can be seen that green product innovation had a significant positive effect on sustainable competitive advantage (standard β = 0.224, *p* < 0.001), and the effect of green relational capital on sustainable competitive advantage changed from 0.290 to 0.259 (*p* < 0.001). H2a was supported. From Model 5, Model 7, and Model 9 in Table 4, H2b was supported.

**Figure 2:**
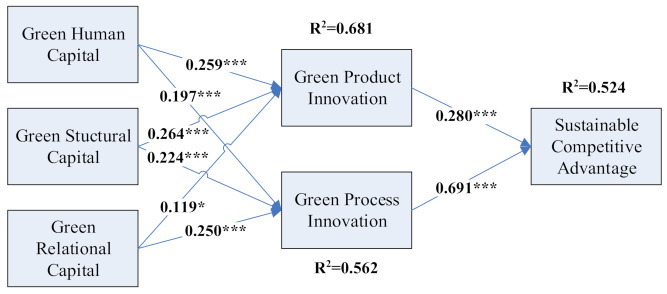
The impact of green intellectual capital on competitive advantage. Note: * Significant at *p* < 0.05 (two-tailed); *** Significant at *p* < 0.001 (two-tailed). The relationships between variables connected by dotted lines were not significant. Standardized solution, *n* = 341. Model fit: χ^2^ (251) = 589.5, comparative fit index (CFI) = 0.995, Root Mean Square Error of Approximation (RMSEA) = 0.014 (90% confidence interval: 0.001–0.023), goodness-of-fit index (GFI) = 0.921, non-normed fit index (NNFI) = 0.994, standardized root mean square residual (SRMR) = 0.049.

We used the structural equation model (SEM) with the bootstrap method to analyze our models. SEM offered an acceptable representation of the data. These antecedents predicted the green product innovation (R^2^ = 0.681), green process innovation (R^2^ = 0.562), and sustainable competitive advantage (R^2^ = 0.524) to a higher extent, respectively. The results are shown in Figure 2.

We also tested the moderation effect. Model 1 to 3 in Table 5 showed that, when the moderating variable environmental leadership entered the regression equation, the interaction terms of green human capital and the moderator had a positive effect on green product innovation (standard β = 0.254, *p* < 0.001). The interaction terms of green structure capital and the moderator had a positive effect on green product innovation (standard β = 0.122, *p* < 0.1). The interaction terms of green relational capital and the moderator had a positive effect on green product innovation (standard β = 0.131, *p* < 0.1). H3a was supported. Models 4 to 6 in Table 5 showed that when the moderating variable environmental leadership entered the regression equation, the interaction terms of green human capital and the moderator had a positive effect on green process innovation (standard β = 0.245, *p* < 0.001). The interaction terms of green structure capital and the moderator had a positive effect on green process innovation (standard β = 0.145, *p* < 0.1). The interaction terms of green relational capital and the moderator had a positive effect on green process innovation (standard β = 0.134, *p* < 0.1). H3b was supported.

Models 1 to 3 in Table 6 showed that when the moderating variable green organizational identification entered the regression equation, the interaction terms of green human capital and the moderator had a positive effect on green product innovation (standard β = 0.255, *p* < 0.001). The interaction terms of green relational capital and the moderator had a positive effect on green product innovation (standard β = 0.275, *p* < 0.001). However, the moderation effect of green organizational identification on green structure capital and green product innovation was not significant. H4a was not supported.

Models 4 to 6 in Table 6 showed that when the moderating variable green organizational identification entered the regression equation, the interaction terms of green human capital and moderator had a positive effect on green process innovation (standard β = 0.123, *p* < 0.1). The interaction terms of green structure capital and moderator had a positive effect on green process innovation (standard β = 0.197, *p* < 0.001). The interaction terms of green relational capital and the moderator had a positive effect on green process innovation (standard β = 0.267, *p* < 0.001). H4b was supported.

**Figure 3:**
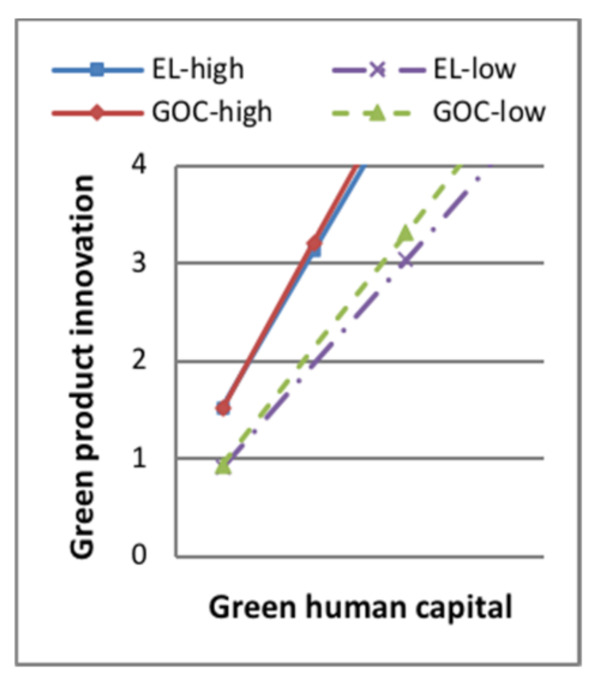
The moderating effect between GHC and GI1.

**Figure 4:**
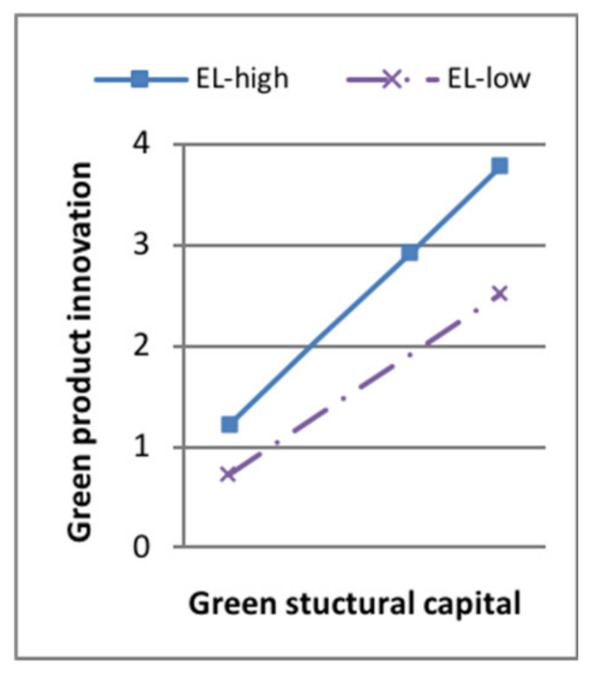
The moderating effect between GSC and GI1.

**Figure 5:**
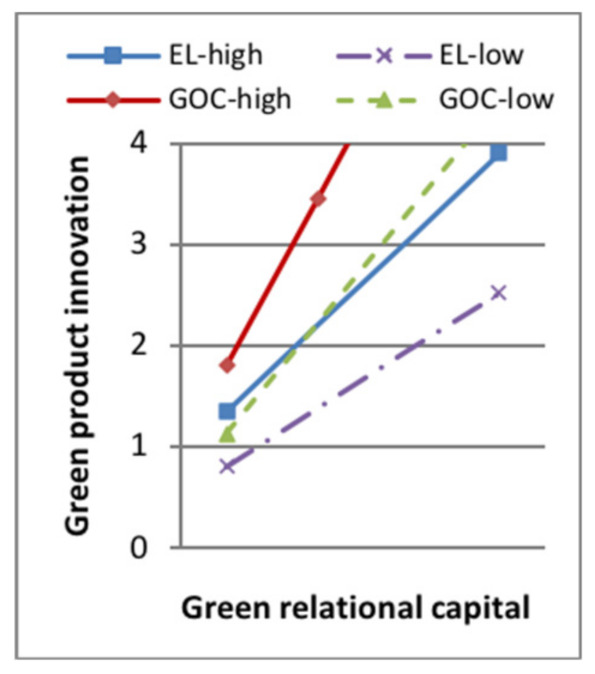
The moderating effect between GRC and GI1.

**Figure 6:**
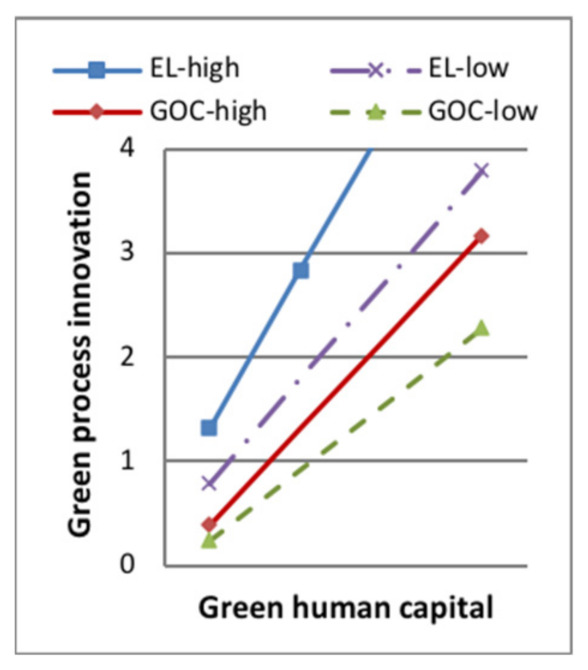
The moderating effect between GHC and GI2.

**Figure 7:**
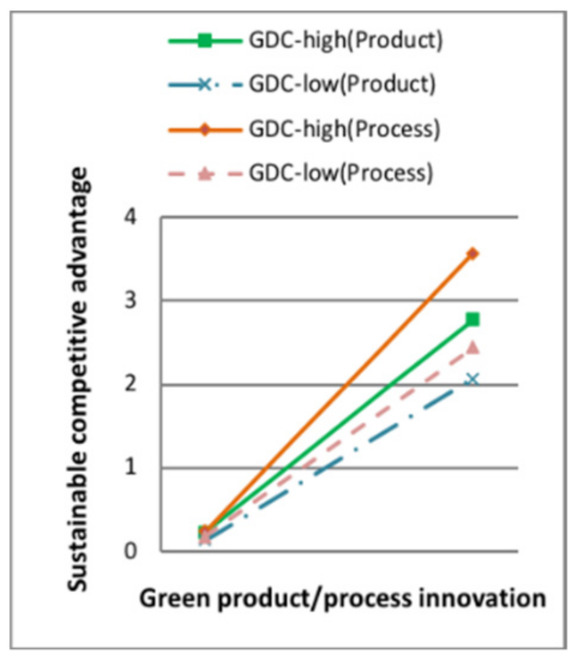
The moderating effect between GI and SCA.

Model 7 in Table 6 showed that, when the moderating variable green dynamic capability entered the regression equation, the interaction terms of green product innovation and the moderator had a positive effect on sustainable competitive advantage (standard β = 0.114, *p* < 0.1). The interaction terms of green process innovation and the moderator had a positive effect on sustainable competitive advantage (standard β = 0.173, *p* < 0.001). H5a and H5b were supported. Some of the moderating effect between green intellectual capital and green product innovation is shown in Figure 3, Figure 4, Figure 5 and Figure 6. The moderating effect between green innovation and sustainable competitive is shown in Figure 7.

We tested the indirect effect by SPSS (IBM, Armonk, NY, USA) bootstrapping macro for moderated mediation [90]. In order to test the moderated mediation effect where three moderators exist simultaneously, we constructed three equations:

1. Gpi_1_ = α_10_ + α_x11_ Gici + α_z12_El + α_xz13_ Gici* El + α_w14_ Goi + α_xw15_ Gici* Goi.

2. Gpi_2_ = α_20_ + α_x21_ Gici + α_z22_El + α_xz23_ GIci* El + α_w24_ Goi + α_xw25_ Gici* Goi.

3. Sca = β_0_ + β_1_ Gici + β_m1_ Gpi_1_ + β_m2_ Gpi_2_ + β_v_ Gdc + β_mv1_ Gpi_1_* Gdc * + β_mv2_ Gpi_2_* Gdc*.

Note: i = 1, Gici = Ghc; i = 2, Gici = Gsc; i = 3, Gici = Grc; Gpi_1_, green product innovation; Gpi_2_, green process innovation; Sca, sustainable competitive advantage; Goi, green organizational identification; El, environmental leadership; Gdc, green dynamic capability.

LLCI represents a lower level of the confidence interval, ULCI represents the upper level of the confidence interval. In the regression of green product innovation, when i = 1, α_xz13_ =0.203 (LLCI and ULCI were between 0.106, 0.300) and α_xw15_ = 0.146 (LLCI and ULCI were between 0.059, 0.234) were all significant. In the regression of green process innovation when i = 1, α_xz13_ = 0.222 (LLCI and ULCI were between 0.122, 0.322) was significant, but α_xw15_ = 0.045 (LLCI and ULCI were between −0.045, 0.135) was not. β_mv1_ = 0.027 (LLCI and ULCI were between 0.008, 0.05) β_mv1_ =0.103 (LLCI and ULCI were between 0.021, 0.184) were all significant. In the regression of green product innovation, α_xw15_ = 0.054 (LLCI and ULCI were between −0.051, 0.159) was not. Others were all significant.

In the regression of green product innovation when i = 3, α_xz13_ = 0.073 and α_xw15_ = 0.270 were all significant. In the regression of green process innovation when i = 3, α_xz13_ = 0.061 (LLCI and ULCI were between −0.057, 0.178) was not significant, α_xw15_ = 0.186 was significant, β_mv1_ = 0.024 and β_mv1_ = 0.094 were all significant.

The study proposed an integrated moderated-mediation effect model where three moderating variables changed simultaneously. The mediating effect was observed in eight cases when the moderating variable was higher or lower than one standard deviation. Table 7 shows the indirect effect under three moderators when green human capital was the independent variable. From the Table 7, we know that if El, Goi, and Gdc were low simultaneously, the mediation of Gpi_1_ or Gpi_2_ was not significant; when El was high, regardless of whether Goi or Gdc was high or low, the mediation of Gpi_1_ or Gpi_2_ was all significant. The tables of the indirect effect under three moderators when the other two green intellectual capitals were independent variables are not displayed. If the three moderators were high simultaneously, the mediation of Gpi_1_ or Gpi_2_ was significant. H6 was supported.

## 5. Discussion

### 5.1. The Mediation Effect for Managerial Implications

Environmental sustainability is now much more important for enterprise sustainable development, and enterprise environmental management is necessary, urgent, but also challenging. NRBV believes that “green” is the key for enterprises to achieve long-term goals [10]. Agricultural enterprises should pay attention to natural resource factors while developing and the balance between enterprise behavior and the natural environment [8]. The main purpose of this study is to explore how can agricultural enterprises become greener by using green intellectual capital to carry out green innovation and establish sustainable competitive advantages and realize “win-win” situations between economic development and environmental protection. The study probed into how agricultural enterprises can use different types of green intellectual capital and different types of green innovation to make better environmental management strategy choices.

Green intellectual capital is a key strategic resource for agricultural enterprises that is valuable, scarce, difficult to imitate, and difficult to replace. Based on the NRBV theory, the study proves that green innovation plays a mediation role between green intellectual capital and sustainable competitive advantage. Pollution provides strong evidence of inefficient use of resources. Green innovation can not only improve the utilization rate of resources, but also reduce pollution [9]. The agricultural enterprises that are the first to carry out green innovation will be compensated with a product premium as the pioneers and will enjoy the first-mover advantage [1]. Green innovation has “double externalities” that are both the positive spillover effects of ordinary innovation and the externalities produced by reducing or eliminating the negative effects on the external environment. Accumulation, application, and management of green intellectual capital can promote agricultural enterprises to carry out green innovation, which not only save production factors and reduce cost but also cause agricultural enterprises to seize the potential opportunities to take the lead in the market and be more efficient and competitive.

When it comes to the three kinds of green intellectual capital and green innovation, first, in the process of green innovation, agricultural enterprises need employees to provide green human capital such as green technology and the knowledge, skills, experience, commitment, and creativity in environmental management [30]. Green human capital applications such as environmental protection knowledge reservation and sharing, green innovation awareness, and green management ability enhancement can promote agricultural enterprises to carry out green product innovation and green process innovation [32].

Second, the environmental management system, green information technology system, environmental protection commitment, green culture, green logo, green brand, green corporate image, and other green structure capital have positive impacts on corporate green innovation. The existing environmental management system can enable agricultural enterprises to break through the original environmental standards and take the initiative to innovate. The green culture creates a good innovation atmosphere for green innovation. Green databases, green patents, green copyrights, green trademarks, and other green structure capital can also support and promote enterprise green product innovation and process innovation.

Third, agricultural enterprises can establish a long-term relationship of trust, commitment, and cooperation with suppliers, customers, partners, and investors by providing green products and services. The establishment of green cooperative relations between them will facilitate the sharing of green knowledge, accelerate the process of green innovation, and promote collaborative innovation. In particular, the establishment of cooperative relations with universities and scientific research institutes will be conducive to the development of green products and green technologies, and ultimately bring sustainable competitive advantages to agricultural enterprises.

In addition, according to the research results, we know that green human capital has a higher impact on green production innovation, and managers should pay more attention to it. Because human capital is embedded in individual employees and is owned by themselves rather than the organization, it will disappear due to the resignation of employees [91]. Enterprise should strive to retain employees with green innovation technology, innovation ability, and provide employees with rewards. Managers could establish incentive systems to reward employees who make special contributions to the development of green ideas and environmental management suggestions. In order to promote green process innovation, a communication platform and knowledge-sharing mechanism could be set up to encourage employees to transform their personal environmental knowledge capital into organizational green intellectual capital, and then into organizational output.

Second, in terms of green structural capital, in order to be more effective in green innovation, agricultural enterprises could optimize the environmental management mechanism, set up specialized environmental protection departments to take responsibility for the green innovation. Agricultural enterprises should pay attention to the information asymmetry between managers and knowledge workers and encourage employees and organizations to form a two-way interactive mechanism through a reasonable incentive mechanism and a performance appraisal method, so that green intellectual capital can break down barriers and flow freely within agricultural enterprises. Agricultural enterprises could introduce a green supply chain management system and establish a green corporate culture and a green agricultural products brand to gain a longer competitive advantage.

Third, in terms of green relational capital, more and more agricultural enterprises choose environmentally friendly suppliers to provide raw materials and semi-products and establish long-term green relations with them. To prolong the competitive advantage, agricultural enterprises can be guided by customers’ demand for green products and extend environmental management to the whole life cycle of products and services, and communicate and cooperate with suppliers, consumers, partners, communities, and scientific research institutions.

Previous studies did not study green intellectual capital in different dimensions, nor did they explore their different impact effects on green innovation. Our study provided different impact results of green intellectual capital on green product innovation and green process innovation. Enterprise should pay attention to the different dimensions of green intellectual capital in the enterprise value platform, and make full use of green human capital, green structural capital, and green relational capital to implement green product innovation and green process innovation, and create greater value for agricultural enterprises to support agricultural enterprises to obtain sustainable competitive advantages.

### 5.2. The Moderation Effect for Managerial Implications

The study proposed that the stronger an enterprise’s environmental leadership and green organizational identification are, the more it can apply green intellectual capital for green innovation, and the stronger its green dynamic capability, the more success of green innovation and the more lasting its competitive advantage will be. However, in the empirical test, we found that the moderation effect of green organizational identification between green structural capital and green product innovation is not significant. This may be due to the infrastructure or process factors of green structural capital, such as green organization design, an environmental management system and a knowledge management system, an operation process, and a control and incentive system all accumulated and modified by agricultural enterprises in long-term production or operation, so green organizational identification has little influence on the relationship between green structure capital and product green innovation.

Although enterprise creates an open, relaxed, and informal innovation cultural atmosphere for green product innovation, in the process, green product innovation may also be influenced by other factors, such as lack of resources, insufficient funds, path dependence, innovation inertia, and difficulty for agricultural enterprises to correct non-environmentally friendly behaviors. The existing environmental management system will also form path dependence and reduce the enthusiasm of green innovation. There are other influencing factors, such as no high-level leaders’ support for the green innovation strategy, insufficient innovation consciousness of leaders and employees, low expectations of competitive advantage, and a lack of environmental knowledge and innovation skills of organization members that affect green structure capital’s support for green innovation.

What is different from previous studies is that our study proposed an integrated moderated-mediation effect model that three moderating variables changed simultaneously. So, we can clearly see the role of different variables. From the model, we found that environmental leadership plays the most critical moderating role. The leaders of agricultural corporate should focus more on ecologically values under environmental commitments, the application of environmental resources, and incentives for green innovation [53]. Environmental leadership affects the environmental behavior orientation inside and outside agricultural enterprises [54]. It has positive influences on the organization’s values, commitments, and aspirations to deal with environmental issues, as well as the understanding and perception of environmental strategic behaviors. Companies could inspire this charismatic leadership style to solve environmental problems through communication and cooperation between leaders and followers, and even the exchange of rights beyond their respective power boundaries [92].

Environmental leadership can motivate organization members to identify, and work hard to realize, the long-term vision of ecological and sustainable development shared by agricultural enterprises. Borck et al. (2008) believe that environmental leadership programs can improve corporate environmental performance by setting environmental goals [93]. Responsible leaders need to create organizational cultures that facilitate green behaviors among their employees [94]. Green human resource management can be implemented successfully if top management supports green innovation performance appraisal, recruitment, reward, selection, and training [95]. So, the leaders of agricultural corporates should stimulate employees’ enthusiasm for environmental protection and green innovation, improve the quantity, quality, and accumulation speed of green intellectual capital of agricultural enterprises, and promote the implementation of green innovation.

The empirical results show that green organizational identification plays a critical moderating role in the whole model. On one hand, from the perspective of organizational employees, green organizational identification enables employees to enhance their awareness and identification of environmental responsibility, and to put forward innovative suggestions in the development of environmental products. On the other hand, from the perspective of organization leaders, improving the green organizational identification can promote the organization’s green innovation strategy formulation, green system establishment, and development of green process innovation [55]. Higher green organizational identification can promote agricultural enterprises to constantly seek common environmental beliefs and goals, actively explore the connection between the latest green technologies and the needs of stakeholders, and actively carry out green innovation in product development and production to solve environmental problems [63].

However, the influence of green organizational identification is less than environmental leadership in the integrated moderated-mediation effect model. A shared model is needed to give the agricultural corporate environmental management adaptive behavior. They could build a cognitive framework for environmental protection to enhance their sense of environmental identity, and then guide the green practice of agricultural corporates. Organizational identification has strong path dependence that affects the cognitive level and cognitive context of agricultural enterprises, and further affects the strategic layout and organizational behavior of agricultural enterprises. Agricultural enterprises should break path dependence, establish a common green organizational identification framework that is different from other organizations, and put green innovation into practice.

Through the empirical results, we can see that green dynamic capability has a positive moderation effect between green innovation and sustainable competitive advantage. Facing the complex and changeable external agricultural environment, there is great uncertainty about whether the competitive advantage brought by green product innovation and process innovation can be maintained. The “dynamic” capability enables agricultural enterprises to better adapt to the changing external environment, continuously digest and absorb technological innovation resources and dynamically occupy some unique resources, reduce the risk of green innovation, and improve the success rate of green product innovation and process innovation [23]. The green dynamic capability can even be regarded as the complementary assets of agricultural enterprises, which can positively promote value promotion and better apply the green intellectual capital. Green intellectual capital needs to be integrated and reorganized to improve innovation performance. Green dynamic ability can enhance the integration effect among various elements of intellectual capital, so that green intellectual capital can be better applied to agricultural enterprises’ green innovation through full digestion, absorption, integration, and utilization. Therefore, in the process of building sustainable competitive advantages, organizations should improve their green dynamic ability.

Finally, from the integrated moderated-mediation effect model, the synergistic effect of environmental leadership, green organizational identity, and green dynamic capability is particularly important, and agricultural enterprises should pay attention to the synergistic effect of them. Under high environmental leadership, green organizational identification, and green dynamic capability simultaneously, agricultural enterprises should constantly identify, evaluate, acquire, analyze, integrate, utilize, and share new environmental knowledge and information that is valuable to green innovation, and establish a formal communication network about green innovation within the organization. Agricultural enterprises should identify opportunities and threats related to environmental problems in a timely manner, adjust three types of green intellectual capital application modes, and make innovative decisions and implement innovative strategies according to green market orientation. By promoting the transformation of green intellectual capital into green innovation, agricultural enterprises can improve the effect of green innovation of their products and realize the “win-win” between the economy and the environment. Finally, enterprise should become greener and establish sustainable competitive advantages.

## 6. Conclusions and Limitations

### 6.1. Conclusions

Through the research, we draw the following conclusions. First, the green human capital, green structural capital, and green relational capital of agricultural corporate have a positive influence on sustainable competitive advantage. Green product innovation and green process innovation play a key mediating role in this impact. Agricultural corporates can establish a sustainable competitive advantage through green innovation.

Second, environmental leadership has a positive moderation effect between green intellectual capital and green product innovation or green process innovation. Green organizational identification has a positive moderation effect between green human capital, green relational capital, and green innovation, except for green structural capital and green product innovation. The stronger an enterprise’s environmental leadership and green organizational identification are, the more it can apply green intellectual capital for green innovation. The stronger the corporate’s green dynamic capability is, the more success in green innovation and the more lasting its competitive advantage will be.

Third, from the integrated moderated-mediation effect model, we know that when the environmental leadership was low, no matter how high the green organizational identification or green dynamic capability were, the mediating effect of green process innovation, green human capital, and green process innovation was not significant. It is the same for green structure capital and green innovation (green product innovation and green process innovation). So, environmental leadership plays the most important role for green innovation. When it comes to green structural capital, environmental leadership and green organizational identification both play important roles for green innovation.

This study is helpful for agricultural enterprises to carry out environmental management more effectively and carry out green innovation through the accumulation and management of green intellectual capital, while obtaining a sustainable competitive advantage. Green innovation of agricultural corporate can not only improve environmental performance, but also establish a sustainable competitive advantage, which is a management behavior of “self-interest” and “altruism”. The research results on the environmental management path of agricultural corporate are beneficial to help agricultural corporates continuously improve the technical level of green agricultural products, generate more green agricultural products, adapt to the green agricultural products market, consolidate the market position, and gain sustainability competitive. Ultimately, this will help to improve the speed of green and sustainable development of agriculture.

### 6.2. Limitations and Further Research

Although we developed a framework to enhance agricultural corporate sustainable competitive advantages and provided some meaningful conclusions and insights into environmental management in the paper, there are certain limitations. Based on the NRBV theory, from the perspective of internal competence and organizational cognition, the research integrated environmental leadership, green organizational identification, and green dynamic capability into the overall framework.

Future studies can focus on the external impacts such as the impact of stakeholder environmental pressure on agricultural enterprises’ green innovation behavior. Second, this study selected agricultural enterprises from the agricultural product-processing industry, such as food manufacturing, beverage manufacturing, tobacco processing, and textile manufacturing as our samples. Further research could be extended to other manufacturing industries and be compared with this study. Third, this study has adopted cross-sectional data in the questionnaire to test the hypotheses so that we cannot demonstrate the dynamic change of environmental leadership, green organizational identification, and green dynamic capabilities at different stages. Therefore, future research can focus on the longitudinal study to track different factors and the sustainable development level of the agricultural enterprises during different stages to do dynamic research. We hope that the research results are useful for managers, researchers, practitioners, and policy makers, and contribute to future research as reference.

## Figures and Tables

**Table 1:** Characteristics of agricultural enterprises (*n* = 341).

	Characteristics	*n*	Percentage
Enterprise type	State-owned	85	24.93%
	Non state-owned	256	75.07%
Enterprise scale (The number of employees)	Lower than 100	40	11.73%
	101–500	101	29.62%
	501–1000	139	40.76%
	1001 or higher	61	17.89%
Region	Northeast China region	142	41.64%
	Yangtze river delta region	133	39.00%
	Pearl river delta region	66	19.36%
Industry	Food processing	52	15.25%
	Food manufacturing	54	15.84%
	Beverage manufacturing	42	12.32%
	Tobacco processing	24	7.04%
	Textile manufacturing	47	13.78%
	Wood processing	31	9.09%
	Furniture manufacturing	49	14.37%
	Rubber products	25	7.33%
	Paper making and paper products	17	4.99%

**Table 2:** Convergent validity and reliability.

Variable	KMO	MFL	AVE	CR	Cronbach’s α
Ghc	0.803	0.717	0.642	0.9	0.862
Gsc	0.909	0.678	0.595	0.921	0.901
Grc	0.784	0.720	0.582	0.874	0.818
Gpi_1_	0.824	0.820	0.692	0.9	0.852
Gpi_2_	0.831	0.830	0.729	0.915	0.875
Sca	0.823	0.623	0.508	0.907	0.884
Goi	0.904	0.787	0.654	0.919	0.897
El	0.862	0.725	0.568	0.902	0.873
Gdc	0.934	0.734	0.616	0.928	0.907

Abbreviations: Ghc, green human capital; Grc, green relational capital; Gsc, green structural capital; Gpi_1_, green product innovation; Gpi_2_, green process innovation; Sca, sustainable competitive advantage; Goi, green organizational identification; El, environmental leadership; Gdc, green dynamic capability; MFL, minimum factor loading.

**Table 3:** Descriptive statistics correlation matrix and test of discriminant validity.

	Mean	S.D.	1	2	3	4	5	6	7	8	9	10	11
1 Type	0.300	0.459											
2 Scale	1.060	0.988	0.251 **										
3 Ghc	4.049	1.094	0.308 **	0.129 **	0.801								
4 Gsc	3.739	1.025	0.224 **	0.082	0.210 **	0.771							
5 Grc	3.890	0.986	0.303 **	0.186 *	0.252 **	0.176 **	0.763						
6 Gpi_1_	3.994	1.095	0.279 **	0.097 *	0.246 **	0.198 **	0.184 **	0.832					
7 Gpi_2_	3.990	1.083	0.293 **	0.112 **	0.235 **	0.167 **	0.201 **	0.461 **	0.854				
8 Sca	4.004	0.849	0.557 **	0.357 **	0.383 **	0.285 **	0.375 **	0.338 **	0.334 **	0.712			
9 Goi	4.384	1.014	0.292 **	0.137 **	0.172 **	0.206 **	0.242 **	0.220 **	0.169 **	0.295 **	0.809		
10 El	4.204	1.061	0.172 **	0.013	0.067	0.066	0.058	0.174 **	0.140 **	0.095 *	0.192 **	0.754	
11 Gdc	4.109	1.028	0.164 **	−0.011	0.096*	0.136 **	0.056	0.159 **	0.180 **	0.097 *	0.176 **	0.283 **	0.785
Ave					0.642	0.595	0.582	0.692	0.729	0.448	0.654	0.568	0.616
Cr					0.900	0.921	0.874	0.900	0.915	0.907	0.919	0.902	0.928

Note: Values on the diagonal represent the square root of convergent validity; Values in the columns are the correlations between two constructs. * Significant at 0.05; ** Significant at 0.01.

**Table 4:** The mediation effect of green product innovation and green process innovation.

	Model 1	Model 2	Model 3	Model 4	Model 5	Model 6	Model 7	Model 8	Model 9
Type	0.513 ***	0.559 ***	0.522 ***	0.464 ***	0.471 ***	0.495 ***	0.501 ***	0.456 ***	0.472 ***
Scale	0.174 ***	0.179 ***	0.153 ***	0.178 ***	0.175 ***	0.183 ***	0.180 ***	0.159 ***	0.156 ***
Ghc	0.300 ***			0.249 ***	0.266 ***				
Gsc		0.218 ***				0.174 ***	0.193 ***		
Grc			0.290 ***					0.259 ***	0.262 ***
GPi_1_				0.195 ***		0.223 ***		0.224 ***	
GPi_2_					0.148 ***		0.179 ***		0.164 ***
Adj-R^2^	0.545	0.511	0.539	0.576	0.562	0.552	0.537	0.582	0.561
R^2^ Change				0.031	0.017	0.041	0.026	0.043	0.022
F Value	136.921 ***	119.357 ***	133.769 ***	116.347 ***	110.165 ***	105.721 ***	99.505 ***	119.362 ***	109.637 ***

Note: *** Significant at 0.001.

**Table 5:** The moderation effect of El regression results.

	Model 1	Model 2	Model 3	Model 4	Model 5	Model 6
Type	0.168 **	0.231 ***	0.227 ***	0.210 ***	0.272 ***	0.246 ***
Scale	−0.051	−0.011	−0.027	−0.037	0.002	−0.019
Ghc	0.268 ***			0.233 ***		
Gsc		0.214 ***			0.165 **	
Grc			0.161 **			0.192 ***
El	0.290 ***	0.231 ***	0.257 ***	0.250 ***	0.200 ***	0.220 ***
Ghc* El	0.254 ***			0.245 ***		
Gsc* El		0.122 *			0.145 **	
Grc* El			0.131 *			0.134 *
Adj-R^2^	0.254	0.191	0.173	0.240	0.183	0.185
R^2^ Change						
F Value	24.208 ***	17.043 ***	15.259 ***	22.444 ***	16.225 ***	16.467 ***

Note: * Significant at 0.05; ** Significant at 0.01; *** Significant at 0.001.

**Table 6:** The moderation effect of Goi and Gdc regression results.

	Model 1	Model 2	Model 3	Model 4	Model 5	Model 6	Model 7	Model 8
Type	0.165 **	0.228 **	0.173 **	0.265 ***	0.264 ***	0.204 ***	0.518 ***	0.522 ***
Scale	−0.065	−0.036	−0.055	−0.020	−0.028	−0.204	0.181	0.186
Ghc	0.330 ***			0.265 ***				
Gsc		0.177 **			0.162 **			
Grc			0.170 **			0.212 ***		
Goi	0.280 ***	0.213 ***	0.335 ***	0.072 *	0.200 ***	0.262 ***		
Ghc* Goi	0.255 ***			0.123 *				
Gsc* Goi		*0.077*			0.197 ***			
Grc* Goi			0.275 ***			0.267 ***		
Gpi_1_							0.263 ***	
Gpi_2_								0.222 ***
Gdc							0.045	0.057 *
Gpi_1_*Gdc							0.114 *	
Gpi_2_*Gdc								0.173 ***
Adj-R^2^	0.249	0.174	0.209	0.177	0.187	0.211	0.534	0.528
R^2^ Change								
F Value	23.577 ***	15.367 ***	19.002 ***	15.636 ***	16.684	19.178 ***	78.952 ***	77.096 ***

Note: * Significant at 0.05; ** Significant at 0.01; *** Significant at 0.001.

**Table 7:** The indirect effect under three moderators.

Mediator	El	Goi	Gdc	Effect	BootSE	BootLLCI	BootULCI
Gpi_1_	3.143	3.370	3.081	0.008	0.021	−0.075	0.012
	3.143	3.370	5.136	0.011	0.016	−0.052	0.011
	3.143	5.397	3.081	0.048	0.029	0.008	0.131
	3.143	5.397	5.136	0.067	0.020	0.008	0.090
	5.266	3.370	3.081	0.079	0.035	0.023	0.165
	5.266	3.370	5.136	0.059	0.026	0.019	0.121
	5.266	5.397	3.081	0.146	0.059	0.036	0.271
	5.266	5.397	5.136	0.110	0.039	0.040	0.194
Gpi_2_	3.143	3.370	3.081	0.002	0.008	−0.019	0.016
	3.143	3.370	5.136	0.004	0.019	−0.046	0.030
	3.143	5.397	3.081	0.007	0.012	−0.019	0.036
	3.143	5.397	5.136	0.154	0.025	−0.032	0.068
	5.266	3.370	4.109	0.052	0.030	0.002	0.119
	5.266	3.370	5.136	0.099	0.036	0.040	0.184
	5.266	5.397	4.109	0.062	0.033	0.002	0.134
	5.266	5.397	5.136	0.119	0.038	0.055	0.207

## Data Availability

Data sharing is not applicable to this article.
